# A metadata-driven approach to data repository design

**DOI:** 10.1186/s13321-017-0190-6

**Published:** 2017-01-24

**Authors:** Matthew J. Harvey, Andrew McLean, Henry S. Rzepa

**Affiliations:** 1 High Performance Computing Service, Imperial College London, London, UK; 2 ICT division, Imperial College London, London, UK; 3 Department of Chemistry, Imperial College London, South Kensington Campus, London, SW7 2AZ UK

**Keywords:** Data repository, Metadata-driven, DataCite, Data preview, Mpublish

## Abstract

The design and use of a metadata-driven data repository for research data management is described. Metadata is collected automatically during the submission process whenever possible and is registered with DataCite in accordance with their current metadata schema, in exchange for a persistent digital object identifier. Two examples of data preview are illustrated, including the demonstration of a method for integration with commercial software that confers rich domain-specific data analytics without introducing customisation into the repository itself.

## Background

Turnkey institutional repositories based on platforms such as DSpace [1] were introduced more 10 years ago, with the early applications directed largely towards archival of publication preprints and postprints. The recent increasing requirement for research data management emerging from funding agencies means that the focus is now shifting to the use of repositories as part of the data management processes. More recent data-centric tools such as Figshare [2] and Zenodo [3] reflect these changes. Such services rely on the minting of persistent identifiers or DOIs for the depositions using the DataCite agency [4]. Metadata describing the deposited material is supplied to DataCite and a DOI is returned. An early example of such research data management is illustrated by a DSpace-based project to produce and then 10 years later to curate a library of quantum-mechanically-optimised molecular coordinates derived from a computable subset of the National Cancer Institutes (NCI) collection of small molecules [5]. One feature of the curation phase [6] of the project aimed to explore the capabilities of the DataCite metadata schemas to improve the discoverability of the deposited data. The metadata can then be exploited to create rich search queries [7]. As a result of the experiences gained from this project, we became aware that one limiting factor to the effective use of metadata was the repository design itself. The next stage therefore was to explore whether what we considered the essential requirements for a data repository could be incorporated into a new design. Here we report the principles used to create such a repository and some of the applications in chemistry that have resulted. These principles may in turn assist researchers wishing to deposit data in identifying the repository attributes that can best expose the discoverability and re-use of their data.

## Data repository design features

 Here we describe the requirements we identified for a metadata-driven repository, an instance of which is deployed by the Imperial College HPC Service at https://data.hpc.imperial.ac.uk

**Fig. 1:**
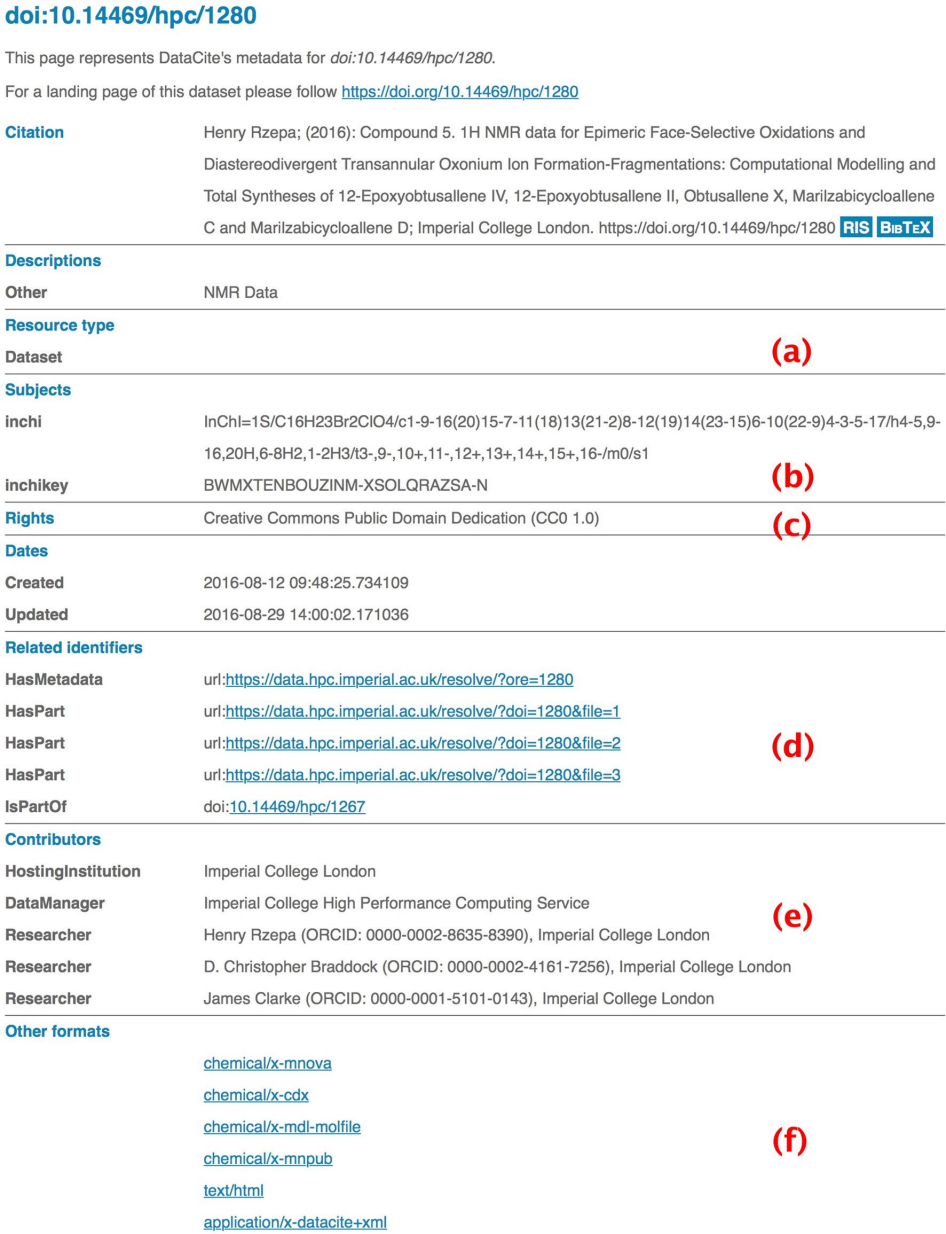
Metadata registered with DataCite for doi:10.14469/hpc/1280, with individual items (*a*)–(*f*) discussed in the section on metadata expression
- The structure of the repository is based on hierarchical collections. Although collections have been a feature of early repositories such as DSpace, relatively little use has been made of them. We first identified the need for such structures from our early project [5] involving individual deposition of >168,000 items. This was deemed necessary since we considered that each item would benefit from having its own unique metadata descriptors, but within the context of a complete collection described using separate metadata. This is illustrated by assigning metadata both to individual entries [9] and to the collection which the individual items are members of [10]. Such hierarchical structures allow a research group to assign collections to project themes and within these to identify sub-collections associated with individual researchers or teams. The sub-collections can be further structured into types of data, other research objects such as software, presentations on the topic and other media such as video. The granularity of this approach is likely to depend very much on the discipline associated with the data. Thus in molecular sciences, the basic object naturally maps to the molecule, since this is the smallest object for which a dataset can be normally be generated and which can usefully be described by its own metadata. It would be less useful or convenient for example to disassemble the molecule into individual atoms as metadata carriers.
  Basing the repository design on collections also reflects the manner in which much modern science is conducted, often via multi-disciplinary collaborations in which each group can generate its own data collections. Collections also greatly facilitate data citation in journal articles. For example, the persistent identifier (DOI) of just the highest collection level of datasets associated with an article can be therein cited, avoiding citation blight. If a particular object (a molecule in our case) is being discussed in the text of the article, it might nevertheless be more appropriate to reference the specific DOI at that stage. Individual citation is also useful in e.g. Tables of results or figures. The metadata for any individual cited dataset will also contain the attribute “is member of”, so that the hierarchy can be both tracked upwards, and via the attribute “has members” downwards (Fig. 1d). This hierarchy also introduces via such metadata further semantics into the citation process itself; each item is placed into appropriate context. Lack of such semantics/context are arguably one of the most deficient aspects of current citation practices in journal articles.
- Our approach to metadata collection is to automate the process whenever possible. In the case of a molecule as an object, there are algorithms which can be used to generate appropriate metadata, the most useful and prominent of which is the InChI (International Chemical Identifier [11]. The task of creating such an identifier is effectively accomplished using the OpenBabel program library [12] or via Javascript-based resources [13]. These can accept as input a variety of chemical documents and generate an appropriate InChI identifier and InChI key uniquely describing them. The repository workflow automatically processes any uploaded data file through this algorithm and records all successful outputs. Such metadata is then associated with the Subject element in the DataCite schema (Fig. 1b).
- Other metadata describing any individual collection or items within the collection can be used to link to other data repositories via the appropriate persistent identifier (DOI) as well as associated journal publications where relevant, again using the DOI. These linkages can of course be made bidirectional by including a citation to the data at the remote site. Such inclusion of bidirectional linking data is currently less automated, but one might envisage future methods for automation involving the ORCID identifier and the ORCID resources as a possible aggregator.
- When a collection or an individual dataset is deposited, the item is immediately issued with a reserved DataCite DOI to allow the authors to quote it in any articles being prepared. Its status is defined as embargoed with an associated access code to allow collaborators to view the item and if necessary to also forward to a journal editor so that they can arrange access for referees. The embargo can be released at a time agreed by the authors, either in advance of the submission of any resulting article, or at the time of open publication of that article. The embargo release is not recursive to any members.
- The repository incorporates an ORE resource map [14], with appropriate metadata descriptors collected to describe the location of this resource map in the repository. This in turn allows a query of DataCite using just the assigned DOI to retrieve the ORE map (Fig. 1d) and facilitates automated retrieval of any individual file contained within a dataset based just on its DOI and if necessary its media type. We have described applications of this procedure termed DOI2Data [15]. Such procedures effectively remove any need to navigate from the landing page associated with the DOI to find and recover data and open up possibilities for large scale automated data mining procedures based just on e.g. top-level collection DOIs. We have also implemented the metadata required to allow the procedure DataCite calls content negotiation [15, 16] (Fig. 1f). An example of date retrieval involving such negotiation might be http://data.datacite.org/chemical/x-mnpub/10.14469/hpc/1280. This queries whether the item with assigned the DOI 10.14469/hpc/1280 has any content associated with the specified media type chemical/x-mnpub and if so retrieves the first instance of such data. If there are multiple such instances in the dataset, then the ORE [14] (or METS) [15] method must be used to select them.
- An emerging feature of data repositories is data preview which can be used as a navigational metaphor. When repositories were largely focused on storing journal articles, preview of the most common document type, the PDF format, was the most important requirement. Most data however is not (certainly should not be) contained in such a document. Clearly, data preview is going to be largely dependent on the discipline associated with the data and it will be difficult to generalise such procedures. We will describe two specific implementations of preview below, but it is important in the initial design of a repository to recognise the need for such rich preview.
- The repository is designed to be operable through a command line and programmatic web API. This allows scripted integration of the deposition process into other workflows such as electronic laboratory notebooks [17].
- The repository to be integrated with the widely-used source code management website Github, and can automatically allocate DOIs to software releases made through that platform. This extends the benefit of DOI citability to software projects without requiring additional effort on behalf of the developer, once the initial configuration has been made.
- The repository is registered via the registry of research data repositories [18]. This involves populating a schema template provided by re3data with the appropriate attributes, which is then processed to create a repository record. This results in the metadata describing the repository itself being assigned a DOI [19]. The repository schema is available as an XML file [20], with further data and metadata information deposited for inspection [21].

- In our design, we have focused on enhancing the FAIR [8] attributes of the data. The first attribute F means the data must be findable and practically this means making the metadata descriptors as rich and complete as possible to enable this. A = Accessibility is achieved by assigning persistent identifiers to the datasets and again associating them with appropriate metadata to enable automated retrieval processes if appropriate. This in turn helps ensure that the data can be accessed in a standard manner to enable its inter-operability in various software environments. R = Re-usability is related to understanding and trusting its provenance and the license terms under which it can be processed.
- The provenance of the deposited data is established from the unique ORCiD identifier of the depositor(s). On the first occasion that the repository is used after initial institutional-based authentication, a redirection to the ORCiD site occurs. There the depositor creates an account or authenticates an existing account, followed by authorising the repository request. The retrieved ORCiD is then added to the metadata manifest for the deposition as a depositor attribute. This initial depositor can then add further ORCiDs as co-authors to the entry; these again are validated automatically from the ORCiD site. This information is then collected and sent to DataCite for aggregation (Fig. 1e).

## Engineering

The repository is intended for use by affiliates of the deploying hosting institution. Deposition first requires requires authentication performed against an institutional authentication and authorisation (A&A) LDAP service. As a matter of policy, the repository also requires the depositing user to provide their ORCID identifier, obtained via an Oauth transaction [22] with the ORCID web service.

The repository is accessed via interfaces designed to function both as a human-friendly UI (accessed via a web browser) and as a programmable API. The latter is essential for integrating deposition into higher level tools and workflows and exposes all the capabilities of the repository. In order to deposit, command-line tools or other programmes using the API must also authenticate and the repository is able to provide delegated access to a user’s account for such tools through a transaction similar to Oauth. This allows automated use performed by a third party tool on behalf of a user to be clearly delineated from actions performed by the user themself and futhermore allows selective revocation of access to the third-party. Current integrations include a computational science portal which manages the execution of quantum chemistry calculations on Imperial College HPC resources. This portal is able to directly publish results into the repository, automatically passing on dataset data files and descriptive metadata.

Data files stored within a repository are maintained on a local filesystem on the server hosting the repository. As data burdens grow to multi-terabyte levels, we expect to migrate this data to remote filesystems. The internal database representation of a dataset deposition allows the files to reside on independent web server, in which case the repository will resolve any requests for them to an HTTP redirect. This would facilitate any future extension of the repository to use a third-party storage solution (e.g. Amazon Web Services S3 object store), or a content distribution network.

The repository automatically generates and publishes metadata records conforming to the DataCite Medadata Schema version 4 [23]. The metadata records are automatically updated whenever a user updates an entry, such as e.g. including a subsequently obtained DOI to a related journal article. At the present time there is a latency of approximately two days before the Datacite search engine index incorporates any updates.

For the Github integration, the repository end-user first associates the repository with Github, again using an OAuth transaction [22]. Thereafter, the repository maintains a list of the user’s Github projects, both public and private, for which DOI creation may be selectively enabled. Once activated, a Github “webhook” [24] is created which automatically makes an HTTP request to the repository whenever a software release is created. This request contains sufficient metadata about the release to allow the repository to create a DOI and automatically populate its metadata. The DOI is recorded within the repository and also added to the release description held within Github.

The repository is implemented in PHP hosted within an Apache web server and depends on a Postgres database. The source code is available on Github [25].

## Metadata expression

The metadata present for a typical deposition conforms to the DataCite metadata schema. Metadata is represented visually in partial form (Fig. 1) and is also available in a semantically more complete form [26]. In addition, each file that is part of a deposited dataset (or is created as a result of the deposition processes) gets registered as a media type. Examples of these formats are shown in Fig. 1f. Specific metadata components are discussed briefly here.

- Resource type identifies whether the item is a dataset or a collection (Fig. 1a).
- Subjects is available for domain-specific information, in this example of unique InChI identifiers and strings [11] derived automatically by parsing the documents in the deposition. The strings subjectScheme and SchemeURI are used to reserve these elements for the subject domain and to disambiguate from similarly named subjects in other domains.
- Related identifiers specifies the location of machine parsable metadata ORE files with use of the ORE resource map being used for the Live Preview described below. The identifiers for HasPart and IsPartOf entries is used to identify the collection hierarchies.
- Contributors includes researchers identified by their ORCID metadata, which in turn allows aggregation by the ORCID organisation.
- Other formats includes domain-specific media types present in the fileset. These entries allow rich searches to be performed, using syntax such as http://search.datacite.org/ui?q=format:chemical/x-* which retrieves all deposited instances of documents assigned the media type chemical/x-cml in all repositories that register the metadata with DataCite.

## The user experience with examples of dataset collections, workflows and metadata

**Fig. 2:**
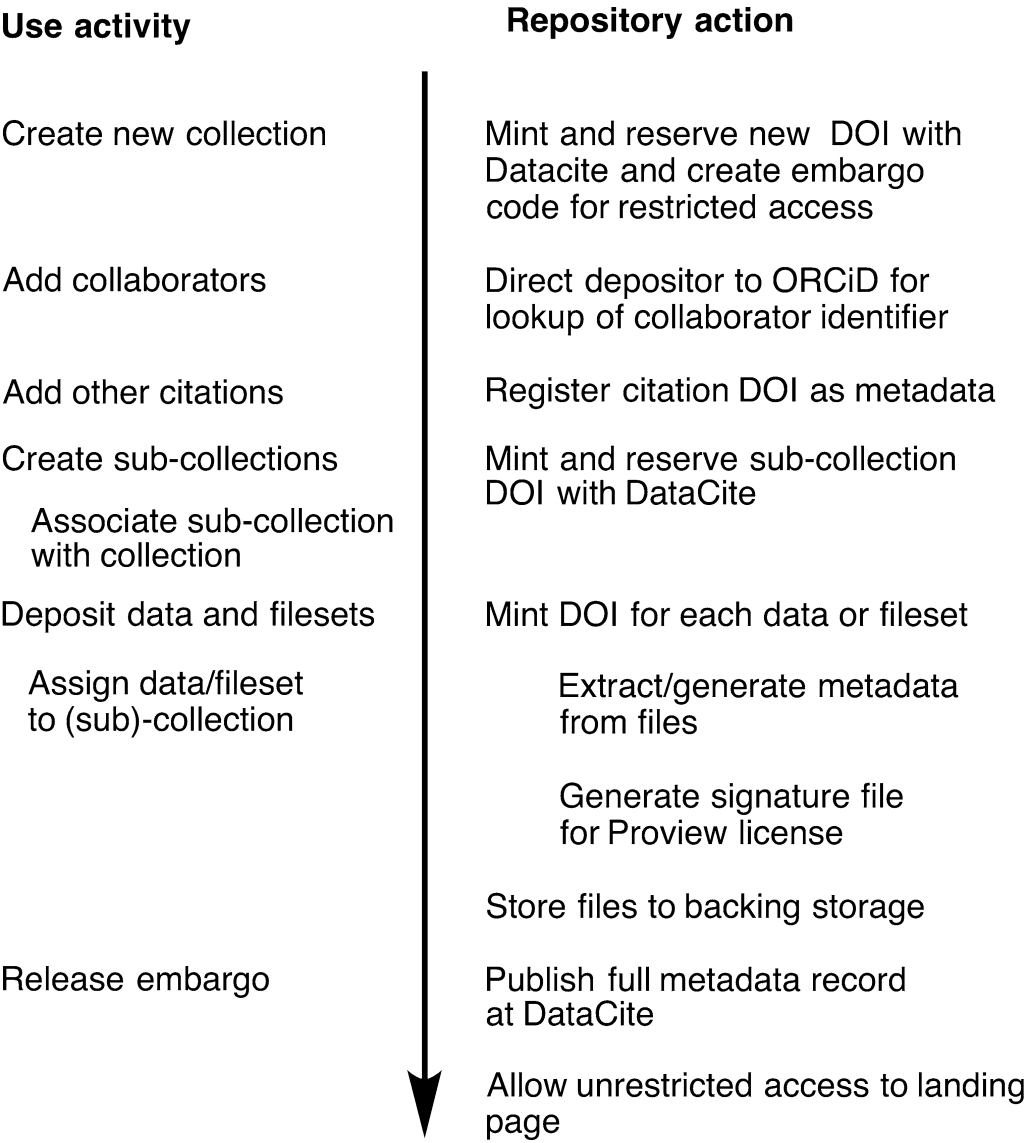
Deposition workflow, illustrating user activity and repository actions

The workflow (Fig. 2) is best illustrated using a recent example [27] associated with a published article [28]. Two basic types of data are associated with this publication; (a) raw and processed instrumental data relating to NMR spectra and (b) computational data deriving from e.g. quantum chemical simulations. Each is associated with a different user interface; the former uses the dataset deposition web page of the data repository itself [19] and the latter is injected into the repository using the command line interface as part of the workflow of a separate ELN (electronic laboratory notebook) via selection of the *publish* button associated with individual computational simulations [17].

- Although the ordering of the actions described below is not imposed, it evolved as an efficient procedure by experimentation and we suggest it as a reasonable starting point for less experienced users. The first requirement is to create an overall project collection using the *add collection* option in the repository itself. For this project it has the DOI:10.14469/hpc/1116. This automatically inherits the ORCiD identifier of the creator, and at this stage all the ORCiDs of the other collaborators can be added as co-authors using the collection *edit* option. Other metadata such as the title and description are also added at this stage; we chose to use the article abstract inherited from the associated journal article as the description in this instance. The final addition of metadata to the master collection relates to *associated DOIs, t*he most important of which is the article associated with the data (DOI:10.1021/acs.joc.6b02008). Also added are others deriving from earlier depositions to other repositories (e.g. DOI 10.14469/ch/191973) which pre-dated the installation of the repository described in this article. A summary of the master collection metadata accruing from these processes can be found at https://data.datacite.org/10.14469/hpc/1116.
- One or more sub-collection(s) are then created to hold e.g. the instrumental NMR data (DOI:10.14469/hpc/1267) and e.g. the computational data (DOI: 10.14469/hpc/1919) These sub-collection pages are edited to make them a *member of* the master collection; reciprocally each sub-collection is identified as a *member* of the master collection. These parent–child relationships are formally defined in the metadata sent to DataCite. The co-authors of sub-collections are not necessarily all the authors of the master collection, but this decision is very much up to the research group to make; in principle each author could be identified by the various contributions they make to the overall project if desired.
- With the basic collection hierarchy now defined, individual datasets can be deposited as and when they emerge from an experiment. We suggest this action is incorporated into the daily laboratory procedures, rather than at the end of any project. For example, when an instrumental data becomes available, the *deposit data* button from the data repository is used. This requires a title and description as metadata, followed by selection of the data files and finally specifying which collection it is a *member of* (in this instance the NMR sub-collection 10.14469/hpc/1267. Some of the uploaded files can themselves serve to help create descriptive metadata about the data. In this instance for every set of molecular specific NMR data, in either raw spectrometer format (Bruker files as a ZIP archive) or in MestreNova (.mnova) format associated with the analysis software being used [29], a separate molecular connection table for that molecule in the form of either a Molfile (.mol) or a Chemdraw file (.cdx or .cdxml) is supplied. If the presence of such a file is detected by the repository workflow scripts, the file itself is passed to OpenBabel [12] in order to generate an InChI string and InChI key which will serve as molecular metadata (Fig. 1b). This exposure of metadata we regard as a better approach in principle to the often used alternative of including image representations of the molecular connectivity, which provides no exposed metadata. Other types of metadata generation could be added to our workflows using other types of content. An example of such a deposition has DOI:10.14469/hpc/1291 for which metadata can again be viewed by pre-pending the resolver https://data.datacite.org.
- The deposition of computational data occurs by a different mechanism, using the computational ELN we have previously described [17]. This system controls the computational workflow, ending with the option to *publish* to pre-selected data repositories, one of which is the one being discussed here. Each entry in this ELN is assigned its own project page. When published, this project becomes mapped to a collection of the same name in the data repository and is initially created in a private embargoed state, requiring an access code to view or edit. We use such inherited collections as holding areas in the data repository, since not all entries may turn out to be suitable for inclusion in the final publication-ready collection. The entries in this holding collection can subsequently be edited to become members of the master or sub-collections at the appropriate point prior to e.g. submission of a manuscript to a journal. An example of such a computational deposition is DOI:10.14469/hpc/1312. In this case it was re-assigned as a member of the master collection 10.14469/hpc/1116 rather than the holding collection inherited from the ELN.
- The final type of dataset was added as a *member of* 10.14469/hpc/1116 and is described in more detail below as LiveView below.

## Examples of data exposure

### ProView

Data, especially if originating from instruments or algorithms encoded in software, can be highly complex. The data may be distributed across multiple data files (around 70 for the datasets described below). Some of these may even be binary-encoded with internal structures that can be poorly documented or hidden for proprietary reasons. Here we describe one example for the processing and re-use of such datasets by non-specialists for whom reliable or rich open-source software solutions may not be available and for whom permanent licensed access to the commercial software may not be practical or cost-effective. The datasets in this example originate from commercial NMR spectrometers and require specialist software to convert the data (in the so-called time domain) into visual representations of the data in the frequency domain (“NMR Spectra”). The raw instrumental outputs take the form of a number of separate data time-domain data files, many of which are without even the meta-information of filename extensions. Without the context of the appropriate software such datasets are essentially inaccessible.

**Fig. 3:**
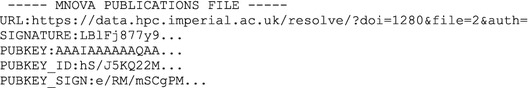
An example auto-generated mnpub file with components containing the URL of the signed resource, the signature, and the identity of the signing entity being the cryptographic key associated with the MestreNova publisher license granted to the repository

MestreNova [29] is commercial software allowing access to such NMR datasets and requires a license entitlement to activate its full feature-set beyond an initial trial period. However, an unlicensed version of MestreNova can have its full function enabled per dataset provided that dataset has been cryptographically signed. These signatures may only be produced by an agency in possession of a MestreNova Publisher license and accompanying signing keys. We have integrated such MestreNova publication into the deposition process of our repository, seamlessly conferring on any NMR dataset deposition the ability to be processed by the MestreNova software. When an NMR dataset in the form of a compressed zip archive or a MestreNova wrapping of such data is deposited into the repository, it is automatically signed, producing a MestreNova-specific “mnpub”-format file which is added to the deposition fileset. This plain-text file contains the URL of the copy of the originating MNova/ZIP file within the repository, along with the cryptographic signature (Fig. 3). When the mnpub file is loaded into an unlicensed version of MestreNova, the associated resource is loaded from the embedded URL and, provided the cryptographic signature validates, the full features of the software are enabled.

We believe this feature demonstrates a powerful incentive for using the repository. By enabling the use of custom software on submitted datasets, the repository becomes more than a passive silo for data, actively enabling depositors and viewers to interact with datasets in a rich, domain-specific way. Furthermore, it is accomplished without the need to develop format-specific enhancements into the repository itself.

### LiveView

The most generic solution to data preview is the deployment of HTML in conjunction with appropriated visualisation routines. One example of this can be illustrated with the following components.

**Fig. 4:**
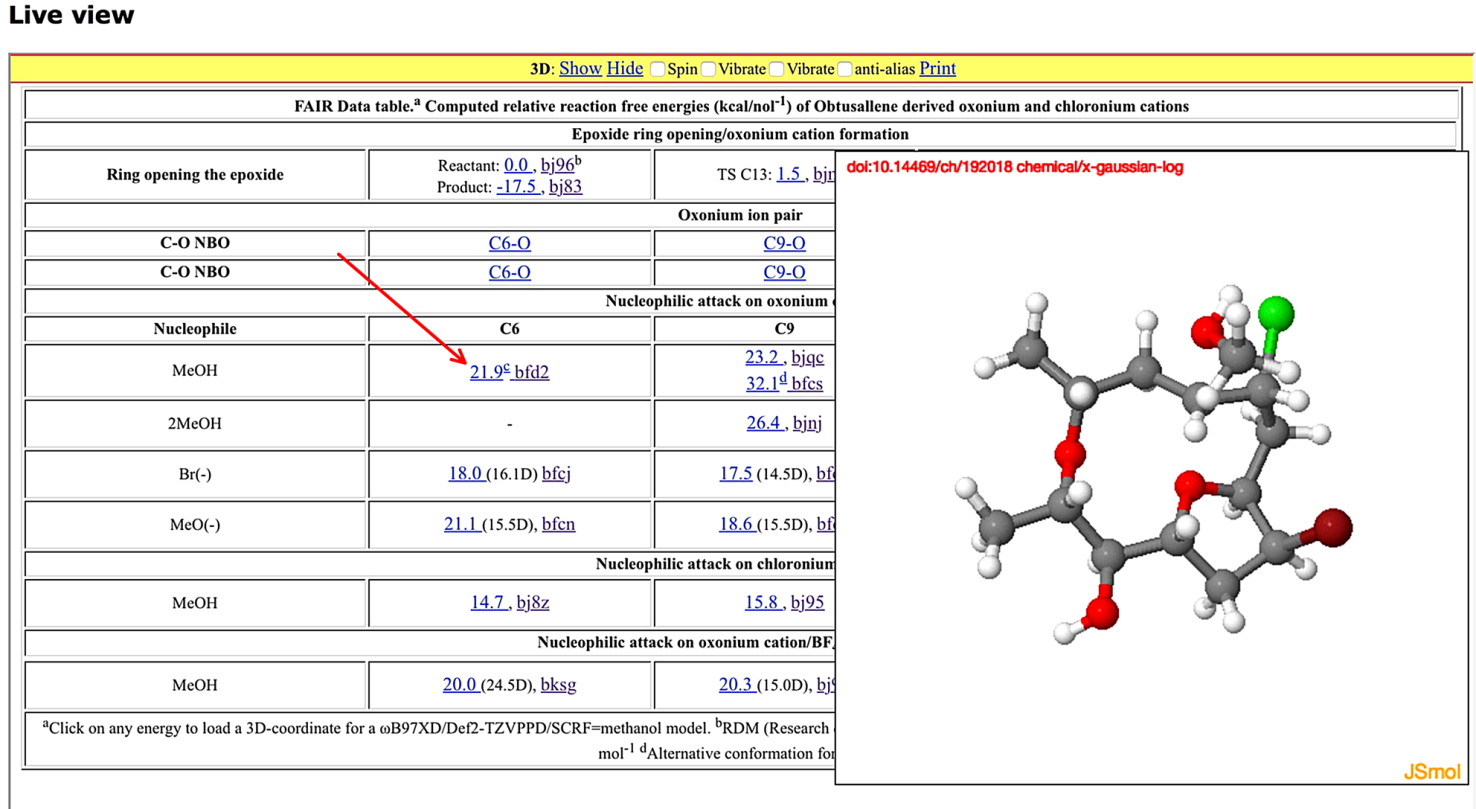
Liveview [31] of a dataset collection expressed using HTML and integrated visualisation package with data retrieved by script-driven DOI-based data retrieval
- The document index.html is itself available for download from the repository and hence can act as a useful template for other authors.

- The HTML document is assigned the reserved name index.html.
- When a document with this name is deposited, it is automatically transcluded into the landing page of the deposition using an HTML iframe:
<iframe name=“liveview” src=“/resolve/?doi=1248&file=13&access=“ width=“100%” height=“600”></iframe>
  where the string doi=1248&file=13&access= references the appropriate database entry for the object assigned the DOI: 10.14469/hpc/1248
- The preview functionality is then enabled by author-specified inclusion of javascript containing utility functions hosted on the repository into the index.html document
<script src=“ https://data.hpc.imperial.ac.uk/js/utilities.js “>
  These serve to invoke an open-source molecular visualiser JSmol [30] which as the name implies is based purely on JavaScript.
- A further script is loaded at this stage, along with a formatting stylesheet:
<script> insertFile(“resolve-doi.js”); insertFile(“table.css”); </script>
  The resolve-doi.js script invokes procedures which accept a dataset DOI as input. Querying the metadata associated with that DOI using the form e.g. http://data.datacite.org/10.14469/ch/192018 allows the path to the ORE or METS resource manifests to be identified (https://spectradspace.lib.imperial.ac.uk:8443/metadata/handle/10042/196268/ore.xml in this instance) and parsing of this manifest then allows the direct path to the data to be extracted and passed through to the JSmol visualisation script.
- An author-initiated entry of the following type in the index.html document then conflates these various actions, the result being a live view of the retrieved dataset in the browser window (Fig. 4): javascript:handle_jmol(‘10.14469/ch/192018’,’;display script;’)”>anchored text</a>

The metadata-driven approach described here is directed towards enabling the FAIR [8] attributes of the data. Registering rich metadata associated with e.g. a primary research object; in molecular sciences the molecule, in turn allows rich searches (the F of FAIR) to be constructed using the generic resources available at the metadata aggregator, DataCite. This approach makes for an interesting contrast with that adopted by e.g. the publisher Elsevier [32] for their recently introduced DataSearch site. This initially appears to be based on the content provided by their journal base, ScienceDirect. The metadata here is simply the likely (but not guaranteed) presence of data within containers such as images or tables. In this approach, a user-driven data search culminates in the user being directed to the data source, which is in fact the article itself as an object. It is then very much up to the user to identify the data of interest within the article, whether in the text or images of the article itself or any associated supporting information. Such an approach is clearly not based on metadata describing data objects such as e.g. molecular entities and leaves the burden on the user to identify and extract any such information themselves. It would be itself fair to suggest that such a process does not fully adhere to the principles of FAIR data.

A clear emerging trend is that journal publication is starting to be associated with procedures for identifying associated data as a primary research object in its own right. The extent to which such data is rendered fully open, in the sense of being compliant with all of the FAIR principles, remains uncertain. It seems likely that journal publishers, who will retain full control over the complete workflows involving data, may not necessarily wish to expose the data as openly FAIR or at the granularity which may be most useful to the researcher. Here we have outlined an alternative metadata-driven mechanism for achieving finely-grained FAIR data exposures in association with journal publication which can be utilized by authors themselves as the creators of research data, and where authors can retain control over the type of metadata captured. In this alternative model, open FAIR data is published at the research institutional level and the associated metadata aggregated at the global level by agencies such as DataCite without a need for intervention by journal-publisher workflows.
